# Determinants of Rehabilitation Follow-Up Non-Adherence Among Stroke Patients in Sibu, Sarawak

**DOI:** 10.21315/mjms-12-2025-865

**Published:** 2026-02-28

**Authors:** Nurul Raihana Rahim, Rou Chen Jee

**Affiliations:** Department of Rehabilitation Medicine, Hospital Sibu, Sibu, Sarawak, Malaysia

**Keywords:** stroke rehabilitation, epidemiology, Sarawak central zone, Hospital Sibu, stroke

## Abstract

**Background:**

Sarawak's vast geography, with remote communities and limited transport infrastructure, poses significant challenges for stroke rehabilitation, yet data on patient attrition in rural Borneo remain limited. This study aimed to evaluate the characteristics of stroke survivors referred for rehabilitation at Hospital Sibu and to identify independent predictors of non-adherence at follow-up.

**Methods:**

Inpatient referrals to the Rehabilitation Medicine Department at Hospital Sibu between February 2022 and December 2024 were retrospectively analysed. To avoid competing risk bias, patients who died before their first follow-up were excluded. Factors associated with non-adherence, including functional status, socioeconomic background and travel time, were examined using multivariable binary logistic regression.

**Results:**

The analytic cohort comprised 631 survivors (mean age 56.7 ± 15.1 years). Overall, 50.4% (*n* = 318) defaulted on their first outpatient appointment. Multivariable analysis identified travel time as the dominant barrier: patients living > 30 min from the hospital had a 40-fold higher risk of default (adjusted odds ratio [aOR] = 39.81; 95% confidence interval [CI]: 22.46, 70.58; *P* < 0.001). Functional dependency also predicted attrition (aOR = 0.98 per Modified Barthel Index point; *P* < 0.001). Ethnic disparities emerged after adjusting for geography: Chinese ethnicity was associated with lower default risk (aOR = 0.63; *P* = 0.028), while Melanau ethnicity remained strongly protective (aOR = 0.35; *P* = 0.001).

**Conclusion:**

Post-stroke rehabilitation attrition in central Sarawak is high (50%). Although functional dependency and ethnicity were significant predictors, geographical inaccessibility is the primary driver of non-adherence. Decentralising care is critical to overcoming this logistical barrier.

## Introduction

Stroke is the rapid onset of focal or global neurological dysfunction lasting more than 24 hours or leading to death, with a vascular cause (1). It remains a leading cause of mortality and long-term disability worldwide, but the burden is increasingly shifting to low- and middle-income countries. Although stroke incidence is declining in some high-income nations, Southeast Asia continues to experience rising prevalence and high mortality rates (2, 3).

In Malaysia, stroke is the third leading cause of death. The Department of Statistics Malaysia documented a sharp rise in stroke-related mortality from 3,651 deaths in 2021 to 8,657 deaths only two years later in 2023 with percentage increase in death is 137%, signalling a growing public health concern (4). Epidemiological data indicate a particularly alarming upward trend among adults under 65 years, driven by the increasing prevalence of vascular risk factors (5). Although national stroke epidemiology is well documented (6), data on post-stroke rehabilitation patterns and socioeconomic disparities in East Malaysia remain limited.

The largest state in East Malaysia, Sarawak, faces distinct challenges in stroke care. Due to its vast terrain, its population is dispersed across urban towns, riverine villages and remote rural settlements, and distances to tertiary hospitals are considerable, yet transportation infrastructure is limited. Consequently, patients from remote regions are constrained by high travel costs, limited availability of services outside major urban centres and other logistical barriers to timely acute stroke treatment and sustained access to rehabilitation services, which are crucial to optimise recovery (7).

The relationship between these factors and rehabilitation adherence can be understood within a conceptual framework of health-seeking behaviour. We hypothesised that follow-up adherence is determined by the interplay of need factors (e.g., stroke severity and functional dependency), predisposing factors (e.g., age) and enabling factors (e.g., socioeconomic support). We identified a critical paradox: while severe functional impairment heightens the clinical need for rehabilitation, it simultaneously creates physical barriers to accessing care, particularly for socioeconomically vulnerable patients. This issue is particularly relevant in Sarawak, where travel times can vary significantly due to riverine geography.

However, limited evidence exists to quantify how these functional and socioeconomic determinants interact to influence attrition among stroke patients in Sarawak. Therefore, the primary objective of this study was to identify independent predictors of non-adherence to outpatient follow-up, based on the demographic, clinical and socioeconomic characteristics of local stroke patients referred for rehabilitation to a representative centre. The findings are intended to inform targeted strategies for strengthening continuity of care in resource-limited and geographically challenging settings.

## Methods

### Study Design and Setting

This study retrospectively analysed all stroke patients admitted to the Rehabilitation Medicine Department of Hospital Sibu, Sarawak, between 17 February 2022 and 31 December 2024. Hospital Sibu is a state tertiary referral centre offering neurology, neurosurgery and rehabilitation services to a predominantly rural catchment population in central Sarawak. This study was approved by the Malaysian Ministry of Health Medical Research and Ethics Committee (NMRR ID-25-00908-DVC).

### Study Populations and Selection Criteria

**Figure 1:**
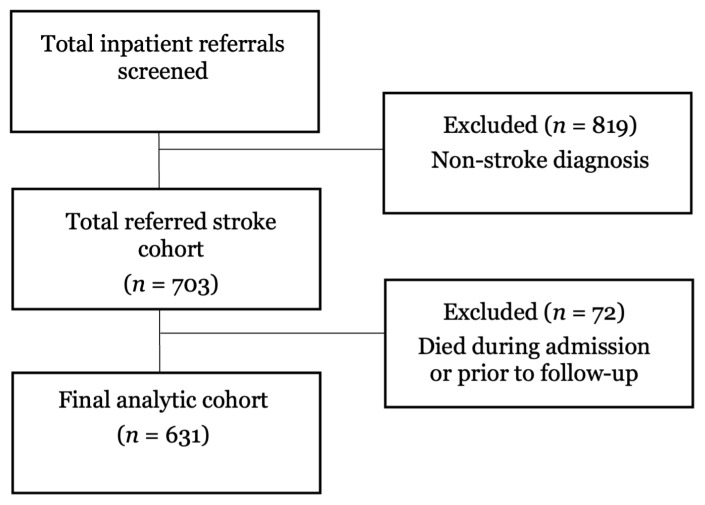
Study flow diagram

The patient selection process is detailed in Figure 1. A total of 1,522 inpatient referrals were screened through a three-stage review of departmental census logs and medical records. The study size was determined by the universal inclusion of all eligible stroke referrals within the 34-month study period; therefore, no prior sample size calculation was undertaken. Of these patients, 703 met the inclusion criteria: (i) confirmed diagnosis of ischaemic or haemorrhagic stroke, and (ii) referral to rehabilitation medicine during the index admission. Both first-ever and recurrent stroke cases were included, and patients referred for non-stroke conditions were excluded.

### Cohort Definition and Mortality

To ensure accurate estimates of rehabilitation adherence and to avoid competing risk bias, two groups were defined:

- Total referred cohort (*n* = 703): comprised all eligible admissions, including in-hospital deaths, to describe the baseline epidemiology; and
- Analytic cohort (*n* = 631): excluded 72 patients (10.2%) who died during the index admission or before their first scheduled follow-up. This survivor cohort was used for all comparative analyses.

### Data Collection and Variable Definitions

Data were extracted using a standardised collection form. They were complete for all variables included in the final analysis. For travel time calculations, residential addresses were verified against hospital registration records for all patients, including defaulters, ensuring no missing values. Variables were defined as follows:

- Demographic and clinical characteristics: Variables included age (stratified as ≤ 20, 21 to 49, 50 to 65, and ≥ 66 years), sex, ethnicity and stroke type. Ischaemic strokes were further classified according to the Oxfordshire Community Stroke Project criteria where documented. Stroke severity on admission was assessed using the National Institutes of Health Stroke Scale (NIHSS) and categorised as minor (0 to 4), moderate (5 to 15), moderate-to-severe (16 to 20) or severe (21 to 40). Comorbidities (hypertension, diabetes mellitus, dyslipidaemia, atrial fibrillation, ischaemic heart disease, smoking status and prior stroke) were recorded from medical documentation.
- Functional status: Trained rehabilitation personnel evaluated functional recovery using the Modified Barthel Index (MBI) at discharge and at the first follow-up (approximately one-month post-discharge). Scores were categorised into standard dependency levels: severe-to-total (0 to 49), moderate (50 to 74) and mild-to-minimal dependency (75 to 100).
- Socioeconomic status (SES): SES was categorised based on formal hospital payer status: (i) B40 (patients registered with Skim Peduli Kesihatan for the B40 [PeKa B40], Social Welfare or fee exemptions); (ii) Government-supported (civil servants and retirees); (iii) Social Security Organisation-supported (private-sector employees with social security coverage); and (iv) Self-funded (patients paying out of pocket).
- Geographical accessibility: Accessibility was defined by travel time rather than physical distance, to account for riverine geography and road conditions. Patients were dichotomised into those living within a ≤ 30-min travel radius and those requiring > 30 min to distinguish the immediate urban catchment area from rural or remote zones.
- Outcome measure: The primary outcome was non-adherence (default), defined as failure to attend the scheduled one-month post-discharge clinic appointment without subsequent re-engagement during the study period.

### Statistical Analysis

Descriptive statistics were used to summarise cohort characteristics. Categorical variables were presented as frequencies and percentages, while continuous variables were expressed as means ± standard deviations. Differences in baseline characteristics between the follow-up and default groups were assessed using Pearson’s chi-square test for categorical variables and independent *t*-tests for continuous variables. Multivariable binary logistic regression was performed to identify independent predictors of non-adherence. The dependent variable was follow-up status, coded as 0 (attended) or 1 (defaulted). Covariates entered into the model included age, sex, ethnicity, stroke type, admission NIHSS, discharge MBI, socioeconomic status and travel time (> 30 min vs. < 30 min). Results are reported as adjusted odds ratios (aOR) with 95% confidence intervals (CI). Statistical significance was set at *P* < 0.05. Data analysis was conducted using SPSS version 26 (IBM Corp, USA).

## Results

### Demographic Characteristics

The analytic cohort of 631 survivors had a mean age of 56.7 ± 15.1 years, and 59.1% were male. The ethnic composition reflected the demographics of central Sarawak, with Iban (43.9%) as the largest group, followed by Chinese (30.1%) and Malay (12.7%). Haemorrhagic strokes were slightly more common (52.9%) than ischaemic strokes (47.1%). Most of the patients with ischaemic stroke were classified as Total Anterior Circulation Infarcts (39.2%) or Lacunar Infarcts (24.9%). Hypertension was the most prevalent risk factor (77.2%), followed by dyslipidaemia (45.0%) and diabetes mellitus (42.5%).

### Factors Associated with Non-Adherence (Univariate Analysis)

Overall, 50.4% (*n* = 318) of the survivors defaulted on their first outpatient rehabilitation appointment. In the bivariate analysis (Tables 1 and 2), no significant differences in adherence were observed based on sex (*P* = 0.226), ethnicity (*P* = 0.129), marital status (*P* = 0.148) or clinical comorbidities (including hypertension, diabetes and prior stroke; all *P* > 0.05). However, employment status prior to stroke onset was significantly associated with adherence (*P* = 0.011): unemployed individuals accounted for the majority of defaulters (53.5%), while those in white-collar occupations demonstrated the highest adherence. Age was also significantly associated with non-adherence (*P* = 0.008 for age group; *P* = 0.001 for mean age), with patients aged ≥ 66 years showing the highest default rate (60.2%).

Geographic accessibility was a major determinant of adherence (*P* < 0.001). Patients living within a 30-minute travel radius had a high adherence rate of 71.8% (*n* = 293/408). In contrast, patients requiring > 30 min of travel time had a default rate of 91.0% (*n* = 203/223). The crude odds of defaulting were approximately 25 times higher for those living outside the 30-minute radius compared to those living closer.

Significant disparities were also observed in functional status. Defaulters had markedly lower discharge MBI scores than attendees (11.9 ± 22.4 vs. 20.6 ± 26.6; *P* < 0.001). Socioeconomic status was likewise significant (*P* < 0.001): Government-supported patients had the highest attendance (66.0%), while those in the B40 and self-funded categories had default rates exceeding 50%.

### Independent Predictors of Non-Adherence (Multivariable Analysis)

In the multivariable logistic regression model (Table 3), travel time was the strongest independent predictor of non-adherence (*P* < 0.001). Patients living > 30 min from the hospital had approximately 39 times higher odds of defaulting than those living within the urban radius (aOR = 39.81; 95% CI: 22.46, 70.58).

Functional status (MBI) remained a significant independent predictor of adherence (*P* < 0.001). For every one-point increase in the MBI score, the odds of defaulting decreased by 2% (aOR = 0.98; 95% CI: 0.98, 0.99). Interestingly, after adjusting for travel time, Chinese ethnicity was associated with a lower risk of default (aOR = 0.63; 95% CI: 0.42, 0.95), while Melanau ethnicity remained a significant protective factor (aOR = 0.35; 95% CI: 0.19, 0.65). Older age and government financial support, which were significant in the univariate analysis, lost significance in the multivariable model.

Notably, the associations of older age and government financial support with non-adherence, observed in the bivariate analysis, were attenuated in the multivariable model (age: aOR = 1.01; *P* = 0.092). This indicates that age and funding source were not independent predictors after adjusting for functional status and geography, suggesting that their effects were likely mediated by these primary barriers.

## Discussion

### Summary of Principal Findings

This study provides one of the most comprehensive descriptions to date of stroke rehabilitation referrals and follow-up adherence in central Sarawak. The findings highlight a convergence of severe post-stroke disability, socioeconomic vulnerability and marked geographical inaccessibility, resulting in a follow-up default rate of 50.4%. The most striking finding is the “distance-decay” effect: adherence fell sharply from 72% to 9% when travel time exceeded 30 minutes. Although functional dependency and socioeconomic status remained significant, the multivariable analysis confirmed that physical accessibility was the dominant driver of attrition.

### The Primacy of Geographical Barriers

Our findings confirm a severe logistical threshold in stroke rehabilitation. The sharp inflection point at 30 minutes suggests that for dependent stroke survivors, the threshold for accessing care is remarkably low. The fact that 91% of patients living just beyond this radius defaulted indicates that centralised hospital-based services are effectively inaccessible to the rural population, which comprised one-third of the cohort. This distance-decay effect likely reflects the distinctive topography of Sarawak, where a 30-minute journey often entails riverine transport or mixed-road conditions. Consequently, the current centralised model inadvertently excludes the population most vulnerable to long-term disability.

### Functional Status and the “Paradox of Need”

Functional status emerged as the second strongest predictor of follow-up adherence. We identified a critical paradox: 90% of patients presented with severe-to-total dependency (MBI < 50). Yet, these patients, who had the greatest clinical need for rehabilitation were the least likely to return. This suggests that the physical burden of transporting a dependent patient often outweighs the perceived benefit of rehabilitation. Patients with severe disability face mobility limitations and require multiple caregivers for transfer, mirroring findings from other low-resource settings where disability acts as a barrier to rehabilitation adherence. Notably, clinical comorbidities (e.g., hypertension and diabetes) were not independent predictors, indicating that the decision to attend follow-up is driven more by functional capability than by medical complexity.

### Sociocultural and Economic Determinants

Beyond geography and functional status, distinct sociocultural and economic patterns were observed. After adjusting for travel time, Chinese and Melanau ethnicity were identified as significant protective factors against non-adherence. This suggests that cultural determinants—such as strong extended family support systems and potentially higher health literacy—may facilitate better appointment adherence in these communities, independent of distance. Conversely, the higher default risk in the reference group warrants further investigation into whether specific cultural perceptions or unmeasured barriers act as obstacles to care.

However, cultural support alone often cannot overcome financial realities. Low socioeconomic status was a significant determinant of default, with B40 patients comprising 66% of defaulters. Financial strain, reliance on daily wage income and the indirect costs of transport likely limit the ability to prioritise follow-up care. Even self-funded patients demonstrated high default rates, indicating that transport costs and income loss remain major barriers even for those just above the poverty line. These findings align with systematic reviews identifying socioeconomic disadvantage as a consistent predictor of poor post-stroke quality of life and outcomes (8).

### Implications for Policy and Clinical Practice

The convergence of these barriers necessitates a shift in how stroke rehabilitation is delivered in Sarawak.

### Clinical Strategy

Early identification of high-risk patients is critical. Discharge planning must be proactive for those living > 30 min away or with low MBI scores. These patients should receive enhanced caregiver counselling and, where possible, immediate linkage to local welfare support to reduce socioeconomic barriers.

### Tele-rehabilitation

Tele-rehabilitation offers a feasible and scalable solution, supported by evidence showing outcomes comparable to in-person therapy (9). However, its successful implementation requires digital literacy, mobile connectivity in rural areas and cultural acceptability among caregivers.

Future research should incorporate geospatial analysis of travel burden, assessment of caregiver burden, evaluation of the cost-effectiveness of tele-rehabilitation models and longitudinal tracking of long-term functional and quality of life outcomes.

### System Reform

The high attrition rate underscores the need to move beyond a purely hospital-centric model. Post-discharge services remain urban-centred, with insufficient continuity of care. Integrating community-based rehabilitation—including nongovernmental organisation-led models such as Stroke Community Rehabilitation Centre (SCORE) (10–12)—with district hospitals could establish a coordinated hub-and-spoke model suitable for Sarawak’s geography.

### Limitations

This study has several limitations. First, it was retrospective and single-centred, which may limit the generalisability of the findings to populations with different geographical or healthcare access profiles. Second, the analysis was susceptible to referral bias, as it included only patients formally referred to the rehabilitation department; thus, it may not capture the full spectrum of stroke burden in the Sibu district (e.g., patients who died before referral or those with mild deficits discharged directly). Third, follow-up data were unavailable for patients who defaulted, preventing direct assessment of their functional outcomes or recovery trajectories. Despite these limitations, this study highlights critical logistical barriers that are likely relevant to other resource-limited settings in Borneo and Southeast Asia.

## Conclusion

Stroke rehabilitation in central Sarawak is constrained by substantial geographic, functional and socioeconomic inequities. Travel time emerged as the dominant barrier to care, rendering hospital-based rehabilitation effectively inaccessible for rural patients. Severe disability and low-income status were the strongest predictors of follow-up default, overshadowing traditional clinical risk factors. Although patients who accessed continuous rehabilitation achieved meaningful functional recovery, more than half were lost to follow-up, exposing major gaps in continuity of care. Strategies that directly address geographic or socioeconomic barriers are essential to ensure sustained access to rehabilitation services.

## Figures and Tables

**Table 1:** Demographic and socioeconomic characteristics and follow-up status of stroke patients (*n* = 631)

	Follow up *n* = 313 (49.6%)	Defaulted *n* = 318 (50.4%)	*P*-value
**Age (years)**			0.001

Mean ± SD (56.7 ± 15.1)	54.8 ± 14.1	58.6 ± 15.9	

**Age group**			0.008

≤ 20 (*n* = 8, 1.3%)	4 (1.3)	4 (1.3)	
21 to 49 (*n* = 198, 31.4%)	112 (35.8)	86 (27)	
50 to 65 (*n* = 229, 36.3%)	119 (38.0)	110 (34.6)	
≥ 66 (*n* = 196, 31.1%)	78 (24.9)	118 (37.1)	

**Gender**			0.226

Male (*n* = 373, 59.1%)	193 (61.7)	180 (56.6)	
Female (*n* = 258, 40.9%)	120 (38.3)	138 (43.3)	

**Ethnic**			0.129

Malay (*n* = 80, 12.7%)	43 (13.7)	37 (11.6)	
Chinese (*n* = 190, 30.1%)	97 (31.0)	93 (29.2)	
Iban (*n* = 277, 43.9%)	125 (39.9)	152 (47.8)	
Melanau (*n* = 63, 10.0%)	39 (12.5)	24 (7.5)	
Others (*n* = 21, 3.3%)	9 (2.9)	12 (3.8)	

**Marital status**			0.148

Married (*n* = 499, 79.1%)	244 (78.0)	255 (80.2)	
Single (*n* = 99, 15.7%)	57 (18.2)	42 (13.2)	
Divorced/widowed (*n* = 33, 5.2%)	12 (3.8)	21 (6.6)	

**Employment status**			0.011

Unemployed (*n* = 306, 48.5%)	136 (43.5)	170 (53.5)	
Blue collar (manual) (*n* = 245, 38.8%)	125 (39.9)	120 (37.7)	
White-collar (office)*n*( = 77, 12.2%)	50 (16.0)	27 (8.5)	

**Social support**			< 0.001

SOCSO (*n* = 101, 16%)	62 (19.8)	39 (12.3)	
Government (*n* = 47, 7.8%)	31 (9.9)	16 (5.0)	
B40 (*n* = 379, 60.1%)	169 (54.0)	210 (66.0)	
Self-funding (*n* = 104, 7.4%)	31 (9.9)	16 (5.0)	

**Travel time to hospital**			< 0.001

< 30 min (*n* = 408, 64.7%)	293 (93.6)	115 (36.2)	
> 30 min (*n* = 223, 35.3%)	20 (6.4)	203 (63.8)	

Values are presented as *n* (%)

**Table 2:** Clinical characteristics, comorbidities, and functional status (*n* = 631)

Characteristic	Follow up *n* = 313 (49.6%)	Defaulted *n* = 318 (50.4%)	*P*-value
**Type of stroke**			0.698

Ischaemic (*n* = 297, 47.1%)	144 (46.0)	153 (48.1)	
Haemorrhagic (*n* = 334, 52.9%)	169 (54.0)	165 (48.1)	

**Comorbidities**			

Hypertension (*n* = 487, 77.2%)	241 (77.0)	246 (77.4)	0.892
Dyslipidaemia (*n* = 284, 45%)	138 (44.1)	146 (45.9)	0.613
Atrial fibrillation ( *n* = 58, 9.2%)	31 (9.9)	27 (8.5)	0.514
Diabetes mellitus (*n* = 268, 42.5%)	131 (41.9)	137 (43.1)	0.722
Smoker (active/ex) (*n* = 202, 32%)	108 (34.5)	94 (29.6)	0.198
History of stroke (recurrent) (*n* = 128, 9%)	68 (21.7)	60 (18.9)	0.375
Ischaemic heart disease (*n* = 42, 6.7%)	23 (7.3)	19 (6.0)	0.468

**Stroke severity (NIHSS), mean score ± SD**			

8.5 ± 4.3	8.4 ± 4.4	8.7 ± 4.2	0.691

**Functional status (MBI), mean score ± SD**			

16.2 ± 24.9	20.6 ± 26.6	11.9 ± 22.4	< 0.001

**Dependency level**			0.110

Severe (0 to 49) (*n* = 561, 88.9%)	270 (86.3)	291 (91.5)	
Moderate (50 to 74) (*n* = 34, 5.4%)	21 (6.7)	13 (4.1)	
Mild (75 to 100) (*n* = 36, 5.7%)	22 (7.0)	14 (4.4)	

Values are presented as *n* (%)

**Table 3:** Factors associated with non-adherence

Variable	Univariate (crude)		Multivariable (adjusted)	
	Crude OR (95% CI)	*P*-value	Adjusted OR (95% CI)	*P*-value
**Functional status**				

Discharge MBI (per point)	0.98 (0.98, 0.99)	< 0.001	0.98 (0.98, 0.99)	< 0.001

**Age (per year)**				

	1.02 (1.01, 1.03)	0.002	1.01 (0.99, 1.02)	0.092

**Gender**				

Female	Ref.			
Male	0.80 (0.59, 1.10)	0.165	0.92 (0.66, 1.29)	0.632

**Stroke type**				

Haemorrhagic	Ref.			
Ischaemic	1.08 (0.79, 1.48)	0.620	1.17 (0.83, 1.65)	0.381

**Stroke severity (NIHSS)**				

	1.02 (0.98, 1.05)	0.386	1.00 (0.96, 1.04)	0.999

**Ethnicity**				

Iban	Ref.			
Melanau	0.38 (0.21, 0.69)	0.001	0.35 (0.19, 0.65)	0.001
Chinese	0.71 (0.49, 1.03)	0.071	0.63 (0.42, 0.95)	0.028
Malay	0.73 (0.45, 1.18)	0.195	0.79 (0.48, 1.26)	0.380
Others	1.04 (0.42, 2.58)	0.930	1.12 (0.43, 2.92)	0.814

**Socioeconomic**				

Self-funded	Ref.			
Government	0.50 (0.24, 1.03)	0.061	0.51 (0.24, 1.09)	0.082
SOCSO	0.61 (0.35, 1.05)	0.076	0.64 (0.36, 1.14)	0.132
B40	1.20 (0.78, 1.86)	0.404	1.02 (0.62, 1.66)	0.940

**Travel time**				

< 30 min	Ref.			
> 30 min	25.86 (15.57, 42.96)	< 0.001	39.81 (22.46, 70.58)	< 0.001

cOR = crude odds ratio (from univariate analysis); aOR = adjusted odds ratio (from multivariable logistic regression); Ref = reference group; Analysis performed on n = 631 survivors (deaths excluded)
